# Risk of SARS-CoV-2 reinfection: a systematic review and meta-analysis

**DOI:** 10.1038/s41598-022-24220-7

**Published:** 2022-12-01

**Authors:** Luojia Deng, Peiqi Li, Xuezhixing Zhang, Qianxue Jiang, DeAnne Turner, Chao Zhou, Yanxiao Gao, Frank Qian, Ci Zhang, Hui Lu, Huachun Zou, Sten H. Vermund, Han-Zhu Qian

**Affiliations:** 1 Department of Bioinformatics and Biostatistics, School of Life Sciences and Biotechnology, Shanghai Jiao Tong University, Shanghai, China; 2 School of Acupuncture-Moxibustion and Tuina, Shanghai University of Traditional Chinese Medicine, Shanghai, China; 3 Yale School of Public Health, Yale University, 300 George Street, New Haven, CT USA; 4 University of South Florida, Tampa, FL USA; 5 School of Public Health (Shenzhen), Sun Yat-sen University, Shenzhen, China; 6 Department of Medicine, Beth Israel Deaconess Medical Center, Harvard Medical School, Boston, MA USA; 7 Xiangya Nursing School, Central South University, Changsha, China; 8 Present Address: GSK plc, Rockville, MD, USA

**Keywords:** Infectious diseases, Diseases

## Abstract

This meta-analysis aims to synthesize global evidence on the risk of reinfection among people previously infected with SARS-CoV-2. We systematically searched PubMed, Scopus, Embase and Web of Science as of April 5, 2021. We conducted: (1) meta-analysis of cohort studies containing data sufficient for calculating the incidence rate of SARS-CoV-2 reinfection; (2) systematic review of case reports with confirmed SARS-CoV-2 reinfection cases. The reinfection incidence was pooled by zero-inflated beta distribution. The hazard ratio (HR) between reinfection incidence among previously infected individuals and new infection incidence among infection-naïve individuals was calculated using random-effects models. Of 906 records retrieved and reviewed, 11 studies and 11 case reports were included in the meta-analysis and the systematic review, respectively. The pooled SARS-CoV-2 reinfection incidence rate was 0.70 (standard deviation [SD] 0.33) per 10,000 person-days. The incidence of reinfection was lower than the incidence of new infection (HR = 0.12, 95% confidence interval 0.09–0.17). Our meta-analysis of studies conducted prior to the emergency of the more transmissible Omicron variant showed that people with a prior SARS-CoV-2 infection could be re-infected, and they have a lower risk of infection than those without prior infection. Continuing reviews are needed as the reinfection risk may change due to the rapid evolution of SARS-CoV-2 variants.

## Introduction

Since the first case of COVID-19 was reported in the early December 2019^1^, the severe acute respiratory syndrome coronavirus 2 (SARS-CoV-2), which causes COVID-19, has infected 420 million people and has been associated with over 5 million deaths worldwide. The rapid spread of this disease is mainly due to the high efficiency of respiratory transmission and universal susceptibility to the virus in the general population^2,3^. The pandemic may lose its increasing momentum only after a high proportion of population become immune to the virus or develop herd immunity. Individuals can obtain immunity through infection or vaccination^4^. Since the first COVID-19 vaccine was available in December 2020, over 60% of the world population has received at least one dose of a COVID-19 vaccine by middle February of 2022. However, there is significant disparity in access to the vaccine by nation, such as 75% in European Union countries and 17% in African countries^5^. SARS-CoV-2 incidence rates may vary by geographic region, but population infection rates have been continuously increasing globally^6–9^. Breakthrough infections were also reported among vaccinated individuals and reinfections were increasing common^10–12^. Then, an urgent public health question is how likely people are to be reinfected.

Studies have shown that SARS-CoV-2 infection-induced immunity may last at least 5–6 months after infection^13,14^, while some small case studies have shown that repeat infections could occur even within 1–3 months after first infection^15–17^. Little is known about the risk of repeat infection among previously infected individuals^14^. Some studies found that the incidence rate of repeat infection was below one percent^18,19^, while other studies showed a higher reinfection rate^20,21^. Other factors may also contribute to the difference of reinfection rates in the studies among general population: (1) diagnostic criteria. Some studies defined a possible reinfection based on an interval of > 30 days between two positive PCR tests^21^ while other studies used an interval ≥ 90 days ^19^; some studies used non-PCR diagnosis approaches^22,23^; (2) different SARS-CoV-2 prevalence and incidence and dominant circulating variants in the study population^4,20^. The demographic characteristics of study population may also account for reinfection rates to some extent, such as proportions of immunocompromised and elderly participants^19,24–26^ and special groups at high risks such as health care workers^4,27^. To better estimate the risk of reinfection and describe the characteristics of reinfection cases, we conducted a systematic review and meta-analysis of the global literature.

## Methods

### Search strategy and selection criteria

This systematic review and meta-analysis was conducted following the Preferred Reporting Items for Systematic Reviews and Meta-Analyses (PRISMA) guidelines^28^. We proposed two research questions: 1) What is the incidence rate of reinfection among people who have been previously infected with SARS-CoV-2? 2) Is the infection risk of SARS-CoV-2 lower among individuals who have been previously infected than among infection-naïve individuals?

We systematically searched 4 electronic databases PubMed, Scopus, Embase, and Web of Science for publications between January 1, 2020 and April 5, 2021. The search terms included: (reinfection*) AND ("COVID-19" OR "Covid-19" OR "SARS-CoV-2" OR "novel coronavirus" OR "2019-nCov" OR "severe acute respiratory syndrome coronavirus 2") AND (cohort OR follow-up OR "followed up" OR longitudinal). We also manually checked the bibliography of each selected paper for additional studies and searched Google Scholar for additional articles. For studies that did not have enough data, we contacted authors to request data, if appropriate.

Studies were eligible for our meta-analysis if they met the following eligibility criteria:

1. The study included a group of participants who have previously been infected with SARS-CoV-2;
2. The study was published in English;
3. The sample size was no less than 100;
4. The study reported quantitative data that allowed for the calculation of the incidence rate of SARS-CoV-2 infection or reinfection;
5. If more than one study was based on the same cohort, only the study with the largest sample size was included;
6. The study included the outcome of SARS-CoV-2 reinfection which was defined as suspected case or confirmed case based on the definitions by PAHO/WHO^29^, and those reported recurrent or re-positive or reactivation of SARS-CoV-2 infection were excluded. In addition, we also included case reports and case series in our systematic review, but not the meta-analysis. The language was limited to English.

### Data screening

**Figure 1:**
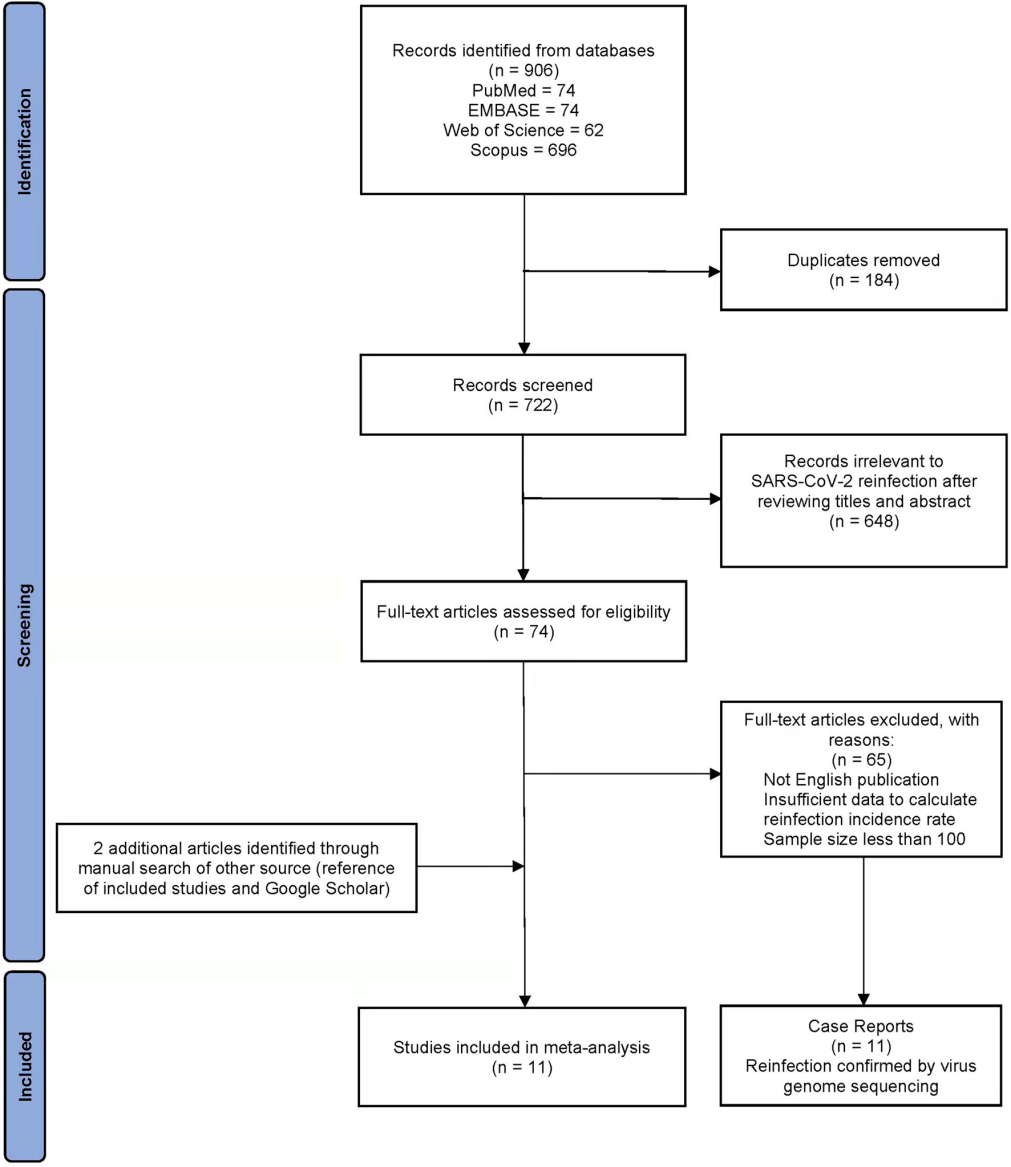
Flow diagram of literature search and study selection.

Four authors (LD, PL, QJ, and YG) performed initial screening independently. Duplicate records were removed using EndNote X9 software^30^. The titles and abstracts were screened to assess whether articles met eligibility criteria. Full texts were assessed if the title and abstract did not provide sufficient information for assessing eligibility. Disagreements were resolved through discussion with the senior investigator (HZQ). Figure 1 describes the literature search and study selection procedures.

### Data extraction and quality assessment

Four reviewers (LD, XZ, PL, and QJ) extracted the data from individual studies independently. Two standardized data extraction forms (Table 1 and Table 2) were used to extract information from the included epidemiological studies and case reports. Data extracted from epidemiological studies included study location, population (general population or health care workers), start and end dates of participant accrual and follow-up, age, sex, cohort follow-up time, laboratory testing of SARS-CoV-2 infection, sample size, and number of new infections. The data on cohort follow-up person-days were either extracted directly from the studies or calculated by multiplying the mean/median follow-up days with sample size. Data extracted from case reports included study country, age, sex, health status other than COVID-19, time interval of two infections, severity and duration of infection and reinfection.

**Table 1:** Characteristics of included epidemiological studies.

Study	Study country & city	Study population	Start and end dates of participant accrual and follow-up	Accumulative cohort follow-up time, person-days		Laboratory testing of SARS-CoV-2 infection	Age mean (SD) or median [IQR]		Sample size		Male sex, n (%)		Number of infections	
				Previously-infected group	Previously-noninfected group		Previously-infected group	Previously-noninfected group	Previously-infected group	Previously-noninfected group	Previously-infected group	Previously-noninfected group	Previously-infected group	Previously-noninfected group
Mumoli 2020^41^	Legnano & Milan, Italy	general		72,360*					804				0	
Xu 2020^42^	Guangzhou, China	general	2020.1.20–2020.4.10	8545.9*		PCR, IgG, IgM	IgG positive 49.1 (14.4) IgG negative 43.2 (12.8)		IgG positive 154 IgG negative 33		IgG positive 68 (44.2) IgG negative14 (42.4)		10	
Abu-Raddad 2021^40^	Qatar	all SARS-CoV-2 cases	2020.2.28–2020.8.12	3,466,461		Nasopharyngeal and/or oropharyngeal swabs PCR			133,266				4	
Hall 2021^4^	England, UK	health care workers	2020.6.18–2020.12.31	2,047,113	2,971,436	Anterior nasal swabs or combined nose and oropharyngeal swabs PCR, sero-Ab	45.6 [34.6–53.8]	45.7[35.8–53.9]	8278	17,383	1425 (17.2)	2585 (14.9)	155	1704
Hanrath 2021^22^	Newcastle upon Tyne, UK	health care workers	2020.3.10- 2020.11.20	179,574*	1,753,701*	PCR, Ab (IgG)	39.5 [30–49]	40 [30–50]	1038	10,137	17.50%	19.50%	0	290
Hansen 2021^23^	Denmark	general	2020.2.26–2020.12.31	1,346,920	62,151,056	Throat swabs PCR			11,068	514,271			72	16,819
Leidi 2021^45^	Geneva, Switzerland	general	2020.4–2021.1.25	178*	5343.8*	RT-PCR naso- or oropharyngeal	46.6 (16.6)	47.3 (16.3)	498	996	242 (48.6)	486 (48.8)	5	154
Lumley 2021^14^	Oxford, UK	health care workers	2020.4.23–2020.11.30	152,983	2,036,358	nasal and oropharyngeal swab PCR, sero-IgG	38 [29–49]	11,276: 38 [29–49] 88: 41 [28–49]	1177	11,364	339 (28.8)	2920 (25.7)	2	26
Masia 2021^26^	Spain	general		26,280*		PCR, Ab (IgG), genome sequencing	Median: 64		146		60.30%		1	
Murillo-Zamora 2021^43^	Mexico	general				PCR			100,432				258	
Pilz 2021^44^	Austria	general		3,116,400*	1,865,984,400*	PCR			14,840	8,885,640			40	253,581

*SD* standard deviation; *IQR* interquartile range; *PCR* polymerase chain reaction; *IgG* immunoglobulin G; *IgM* immunoglobulin M; *Ab* antibody.

*The duration of follow-up was calculated through multiplying sample size by median or mean follow-up time.

**Table 2:** Characteristics of included case reports.

Article	Country	Age, sex	Health status other than COVID-19	Time interval (Date of first laboratory PCR positive-date of first laboratory PCR positive during reinfection)	Severity of reinfection compared with prime infection	Note (e.g., different variants, severity, vaccination history, …)	Duration of reinfection compared with prime infection (duration of prime infection, duration of reinfection) (Diagnostic criterion ‡)
Larson 2020^46^	USA	42, M#	Healthy	65 (2020.03.20–2020.05.24)	More severe	Several potential variations, including one high confidence variation	Longer (10, 14) (Clinical)
Goldman 2020^51^	USA	60 ~ 69, ND	Severe emphysema (FEV1 34% predicted) on home oxygen, and hypertension	140 (ND)	Less severe	Revealed 10 high confidence intra-host single nucleotide variants (iSNVs) of which 5 type the March sequence to clade 19B, and 5 type the July sequence to 20A	ND
Lee 2020^15^	South Korea	23, F	Healthy	26 (2020.03.11–2020.04.06)	Similar	Different SARS-CoV-2 subtype (pike protein D614G substitution, mutations characterizing the clade “V” (ie, nsp6 L37F and ORF3a G251V)	Shorter (15,13) (Laboratory)
To 2020^52^	China	33, M	Healthy	142 (2020.03.26–2020.08.15)	Less severe	The first viral genome belongs to GISAID clade V, Nextstrain clade 19A, and Pangolin lineage B.2 with a probability of 0.99. The second viral genome belongs to GISAID clade G, Nextstrain clade 20A, and Pangolin lineage B.1.79 with a probability of 0.70	Shorter (3,0) (Clinical)
Gousseff 2020^49^	Switzerland	36, F#	Healthy	204 (2020.4.10–2020.10.31)	Similar	Two different SARS-CoV-2 genomes both belonging to clade 20A	Shorter (14,10) (Clinical)
Gupta 2020^47^	India	25, M#	ND	108 (2020.05.05–2020.08.21)	Similar	A genetic variant 22882 T > G (S: N440K) found during reinfection in I2	Longer (8,14) (Laboratory)
		28, F#	ND	111 (2020.05.17–2020.09.05)	Similar		Shorter (12,6) (Laboratory)
Prado-Vivar 2021^16^	Ecuador	46, M	ND	63 (2020.05.20–2020.07.22)	More severe	The first infection variant belonged to clade 20A and lineage B1.p9, whereas the second infection variant belonged to clade 19B and lineage A.1.1	shorter(22, 15)(Clinical and laboratory )
Klein 2021^18^	USA	60 ~ 70, M	Renal transplantation 2 years prior end-stage renal disease	233(ND)	Less severe	The virus genome sequenced from the reinfection had 12 mutations not observed in the virus sequenced from the primary infection	Shorter (27,15) (Clinical)
Van Elslande 2021^17^	Belgium	51, F	Asthma	90 (2020.03.09–2020.06.10)	Less severe	Distinct: the initial infection was caused by a lineage B.1.1 SARS-CoV-2 virus and the relapsing infection by a lineage A. Eleven mutations were identified	Shorter (49,7) (Clinical)
Salehi-Vaziri 2021^48^	Iran	32, F	Healthy	63 (2020.04.20-ND)	More severe	D614G mutation	Longer (28,30) (Laboratory)
		42, M	Healthy	111 (2020.3.10-ND)	Less severe	D614G mutation	Longer (39,5) (Laboratory)
Tillett 2021^50^	USA	25, M	Healthy	48 (2020.03.25–2020.06.05)	More severe	Both specimens were members of clade 20C, but have different mutations	ND

^#^Health care workers; ‡Clinical diagnosis such as presence of duration or hospital discharge, or laboratory results of polymerase chain reaction (PCR) testing.

M—Male.

F—Female.

ND–no data.

The Newcastle–Ottawa Quality Assessment Form for Cohort Studies^31^ was used to appraise the methodological quality (supplementary Table 1). The total score is 9, and the higher score means better quality. An additional criterion was added to assess the quality of laboratory testing of reinfection—whether whole-genome sequencing of SARS-CoV-2 was used to assess reinfection.

During data extraction and quality assessment, discrepancies were resolved through discussion with the senior investigator (HZQ).

### Data analysis

Data analysis was performed using R software (version 4.0.2)^32^ and gamlss (version 5.3.4)^33^, gamlss.dist (version 5.3.2)^34^, meta (version 4.19.0)^35^, metafor (version 3.0.2)^36^, dmetar (version 0.0.9)^37^ packages.

Our main outcome of interest was SARS-CoV-2 reinfection. Two primary analyses were performed to calculate the pooled incidence rate of SARS-CoV-2 reinfection and to compare the pooled incidence rates among those who were previously infected and among those who were never infected with SARS-CoV-2.

The incidence rates were calculated by dividing number of cases by total person-time followed up among these participants. The total person-time for each study was either directly provided or calculated based on the time span and number of participants. To include studies with zero event into analysis, the two-part zero-inflated beta (ZIB) distribution was used to calculate the pooled incidence rate^33,34^. The pooled incidence rate was defined as the marginal mean of ZIB^38^. We also simulated the incidence rate for 1000 times using ZIB distribution with estimated parameters and used the standard errors of these simulated samples to calculate 95% confidence intervals (CI) of the pooled incidence rate.

To compare the incidence rates between previously infected individuals and uninfected individuals, we calculated hazard ratio (HR) by computing the incidence ratios between previously infected and uninfected groups. Heterogeneity was assessed by Cochran’s Q test, and the degree of heterogeneity was assessed by *I*^*2*^ statistics. Due to the high heterogeneity of included studies, we used random-effects models. HRs were pooled with DerSimonian-Laird method^39^ Significance was considered to have a two-sided *P* value < 0.05.

Subgroup analyses were also conducted among health care workers and the general population.

### Registration

The study protocol was registered with the International Prospective Register of Systematic Reviews (PROSPERO) (https://www.crd.york.ac.uk/PROSPERO/, ID: CRD42021265784).

## Results

A total of 906 articles were retrieved from electronic database searches. After removing duplicates, 722 records were retained. After 648 records were excluded through reviewing the titles or abstracts, 74 full texts were reviewed, and 2 additional articles were identified through manual searches of the references and Google Scholar. After review, 11 epidemiological studies and 11 case reports were eligible (Fig. 1).

Of the 11 included epidemiological studies for meta-analysis (Table 1), three were conducted in the United Kingdom^4,14,22^, and one in each of 8 countries Denmark^23^, Qatar^40^, Italy^41^, Spain^26^, China^42^, Mexico^43^, Austria^44^, and Switzerland^45^. All 11 studies were either prospective or retrospective cohort studies published between 2020 and 2021. Length of follow-up for assessing new SARS-CoV-2 infections among participants with exposure to index cases ranged from 3 to 9 months. No study reported vaccination status of study participants. All 11 studies were included in calculating the pooled incidence rate, and 6 were also included in the analysis of comparing previously-infected and uninfected individuals^4,14,22,23,44,45^. Of 11 epidemiological studies, 27% (3/11) were conducted among health care workers and 73% among the general population. A total of 9,711,525 participants in these 11 epidemiological studies were included in meta-analysis, including 271,734 with a history of SARS-CoV-2 infection and 9,439,791 without. The mean age of all participants was 42.6 years.

The pooled incidence rate of SARS-CoV-2 reinfection was 0.70 (standard deviation [SD] 0.33) per 10,000 person-days or 2.5% (1.2%) person-years (Table 3). Subgroup analyses showed the incidence rate was 0.30 (SD 0.18) per 10,000 person-days or 1.1% (SD 0.6%) person-years among health care workers and 0.85 (SD 0.49) per 10,000 person-days or 3.1% (SD 1.8%) person-years among the general population (Table 3). The difference between the two groups was statistically significant (*P* = 0.02) (Table 3).

**Table 3:** Pooled incidence rate of reinfection and subgroup analysis.

	Number of included studies	Number of studies with zero reinfection	Total number of participants	Total number of reinfections	Pooled incidence rate per 10,000 person-days (SD)*	Group difference *P* value
Total	11	3	271,734	547	0.70 (0.33)	
**Subgroups**						
Health care workers	3	1	10,493	157	0.30 (0.18)	0.02
General population	8	2	261,241	390	0.85 (0.49)	

*SD* standard deviation.

**Figure 2:**
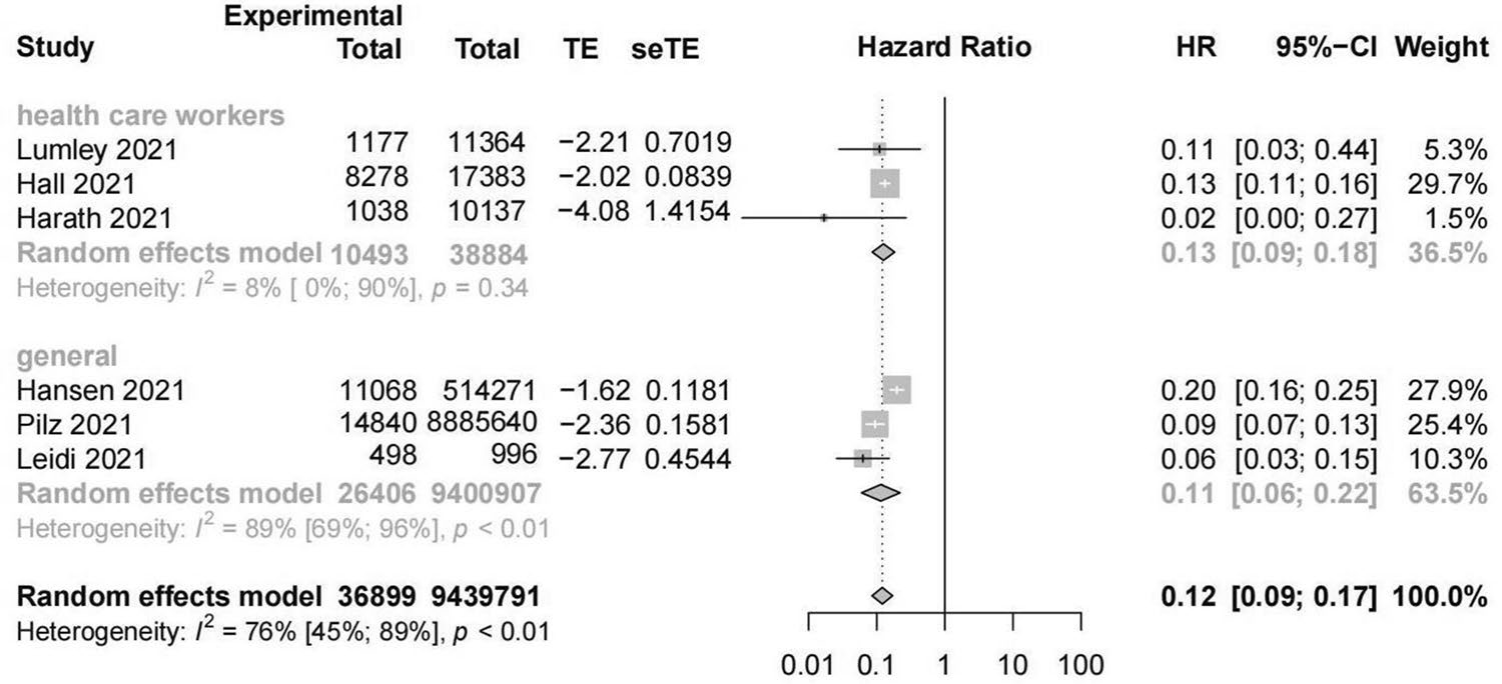
Forest plot of association between previous infection and SARS-CoV-2 reinfection.

A random-effects meta-analysis of six studies reporting new SARS-CoV-2 infection among the previously-infected group and noninfected group showed a significantly lower risk of infection in the previously-infected group (HR = 0.12, 95% CI = 0.09–0.17, number of estimates (k) = 6, *I*^2^ = 76%, 95% CI = 45%-89%). The HR was slightly lower among the general population (HR = 0.11, 95% CI = 0.06–0.22, k = 3, *I*^2^ = 89%, 95% CI = 69%-96%) than health care workers (HR = 0.13, 95% CI = 0.09–0.18, k = 3, *I*^2^ = 8%) (Fig. 2). Influence analysis indicated the robustness of the results (Supplementary Fig. 1). Egger’s test showed no publication bias in these studies (*P* = 0.34).

Nine studies^4,14,22,23,40,42–45^ were ranked as good or fair quality^31^. Two studies^26,41^ were considered as poor quality.

A total of 13 SARS-CoV-2 reinfection cases were reported in the 11 case reports or case series^15–18,46–52^ and all were confirmed via whole-genome sequencing. More than half of cases (7/13) were reported in 2020. Participant age ranged from 25 to 70 years, and about half (7/13) were men. Four cases were health care workers, and three had comorbid diseases (Table 2).

The time interval between two episodes of infection ranged from 26 to 233 days. Five cases had less severe symptoms of reinfection than first infection, four had more severe symptoms, and four had similar severity. Eight cases had a shorter duration of disease in the second infection than the first, while three patients had longer duration in their second infection (Table 2).

## Discussion

This systematic review and meta-analysis summarized the evidence on likelihood of SARS-CoV-2 reinfection. The pooled incidence rate was 0.70 per 10,000 person-days or 2.5 per 100 person-years. People who were previously infected were 87% less likely to get reinfection than those who were never infected (HR = 0.12). Although the risk of reinfection may be low for individuals, the global number of reinfections could be several millions in one year, considering over 440 million people had been infected worldwide by February of 2022. It is suggested that people who have been infected should also receive vaccinations and use personal protections to reduce the risk of reinfection. Given the global number of reinfections, this guidance is warranted.

Our meta-analysis showed that health care workers had lower incidence of reinfection than the general population (0.30 vs 0.85). Health care workers have more COVID-19 exposure than the general population, but they may have higher risk awareness and better use of personal protections than the general population, leading to a lower likelihood of reinfection.

In the results of our analysis, the *I*^2^ statistics and Cochran’s Q test result indicated relatively strong heterogeneity among included studies. The reinfection rates could vary across different study geographic locations and time periods. The extent of local virus spread could also affect the reinfection rates. Such differences may underlie the heterogeneity and cause a significant Cochran’s Q test result in the general population sub-group. Due to lack of data, we cannot control such factors in our meta-analysis.

The risk of reinfection could be affected by numerous factors. For example, the likelihood for a person to get an infectious disease depends on the chance of exposure and use of personal protection, and reinfection is also associated with decline in immunity and virus mutation and circulating SARS-CoV-2 variants^53,54^. Our analysis has limitations. First, the median follow-up time of participants was less than 6 months in most studies included in this meta-analysis. The infection-induced immunity may wane over time, and the risk of reinfection may increase. Studies have found that the vaccine-induced neutralizing antibody response against the spike protein of five major SARS-CoV-2 variants declined over time^55^ , and infection-induced humoral immunity against SARS-CoV-2 (IgG level) might not be long lasting in persons with mild illness^56^. Real-world research is needed to assess and the duration of infection- and vaccine-induced immunity against SARS-CoV-2 reinfection. The duration of immune response may also be moderated by other factors such as age. A study on immunogenicity of an mRNA vaccine showed that serum neutralization and levels of binding IgG or IgA after the first vaccine dose were lower in older individuals, but neutralization against SARS-CoV-2 variants was detectable regardless of age^57^. Second, our literature search was limited to publications before April 5, 2021, when the Omicron variant has not emerged. Studies have shown both immune evasion by Omicron variant contributed to a higher transmission rate than other variants^58–60^ Third, As the vaccination status was not reported in the included studies, vaccination status was not considered in assessing the risk of reinfection. Before April 2021, most countries had not started to vaccinate their populations, or if had started, might still have low vaccination rates. Therefore, the estimated risk of reinfection is unlikely to be significantly confounded by vaccination. Updated meta-analysis is needed to estimate the risk of reinfection in the circumstance of Omicron as the dominant variant. As the data related to SARS-CoV-2 reinfection becomes more available, sub-analyses could be explored to examine the rates of reinfection by a variety of covariates, such as age, sex, comorbidities and history of vaccination. The results from this meta-analysis may serve as a comparison to future research on the risk of reinfection of Omicron and new emerging variants among the widely vaccinated population^61,62^.

## Conclusion

In conclusion, our meta-analysis suggests that there is a risk of reinfection among people who have been previously diagnosed with COVID-19. Vaccination may produce higher neutralizing antibody titers compared to SARS-CoV-2 infection^63^, and people who are infected with SARS-CoV-2 can still benefit from vaccination, particularly for the purposes of preventing more transmissible variants.

## Supplementary Information

Supplementary Information 1.

Supplementary Information 2.

## Data Availability

The data analyzed in this meta-analysis are from previously published studies, which have been cited.
