# Supplementary material for: Risk of SARS-CoV-2 reinfection: a systematic review and meta-analysis
Source: Sci Rep. 2022 Dec 1;12:20763. doi: 10.1038/s41598-022-24220-7 (PMC9714387; doi:10.1038/s41598-022-24220-7)
Supplement: Supplementary file 1 — Supplementary Information 1. [file 41598_2022_24220_MOESM1_ESM.docx]

**Supplementary Table 1. Quality Assessment by Newcastle-Ottawa Scale***

| **Study** | **S-1** | **S-2** | **S-3** | **S-4** | **C** | **O/E-1** | **O/E-2** | **O/E-3** | **Total** | **Genome Sequencing** |
| --- | --- | --- | --- | --- | --- | --- | --- | --- | --- | --- |
| Mumoli 2020[41] | 1 | NA | 1 | 1 | 0 | 0 | 0 | 1 | 4 | No |
| Xu 2020[42] | 1 | NA | 1 | 1 | 2 | 1 | 1 | 1 | 8 | No |
| Abu-Raddad 2021[40] | 1 | NA | 1 | 1 | 1 | 1 | 1 | 1 | 7 | Yes |
| Hall 2021[4] | 1 | 1 | 1 | 1 | 1 | 1 | 1 | 1 | 8 | No |
| Hansen 2021[23] | 1 | 1 | 1 | 1 | 1 | 1 | 1 | 1 | 8 | No |
| Hanrath 2021[22] | 1 | 1 | 1 | 1 | 2 | 1 | 1 | 1 | 9 | No |
| Leidi 2021[45] | 1 | 1 | 1 | 1 | 2 | 1 | 1 | 1 | 9 | No |
| Lumley 2021[14] | 1 | 1 | 1 | 1 | 1 | 1 | 1 | 1 | 8 | No |
| Masia 2021[26] | 1 | 0 | 1 | 1 | 0 | 1 | 1 | 1 | 6 | Yes |
| Murillo-Zamora 2021[43] | 1 | 1 | 1 | 1 | 2 | 1 | 1 | 1 | 9 | No |
| Pilz 2021[44] | 1 | 1 | 1 | 1 | 2 | 1 | 1 | 0 | 8 | No |

NA, not applicable

S-1: Representativeness of the exposed cohort

S-2: Selection of the non-exposed cohort

S-3: Ascertainment of exposure

S-4: Demonstration that outcome of interest was not present at start of study

C-1: Comparability of cohorts on the basis of the design or analysis controlled for confounders

O-1: Assessment of outcome

O-2: Was follow-up long enough for outcomes to occur

O-3: Adequacy of follow-up of cohorts

Items S-1, S-2, S-3 and S-4 are related to study selection in NOS

C has 2 items related to study comparability in NOS

O/E-1, O/E-2, O/E-3 are related to study exposure/outcome measurement in NOS.
