# Supplementary figures and images for: Risk of SARS-CoV-2 reinfection: a systematic review and meta-analysis
Source: Sci Rep. 2022 Dec 1;12:20763. doi: 10.1038/s41598-022-24220-7 (PMC9714387; doi:10.1038/s41598-022-24220-7)

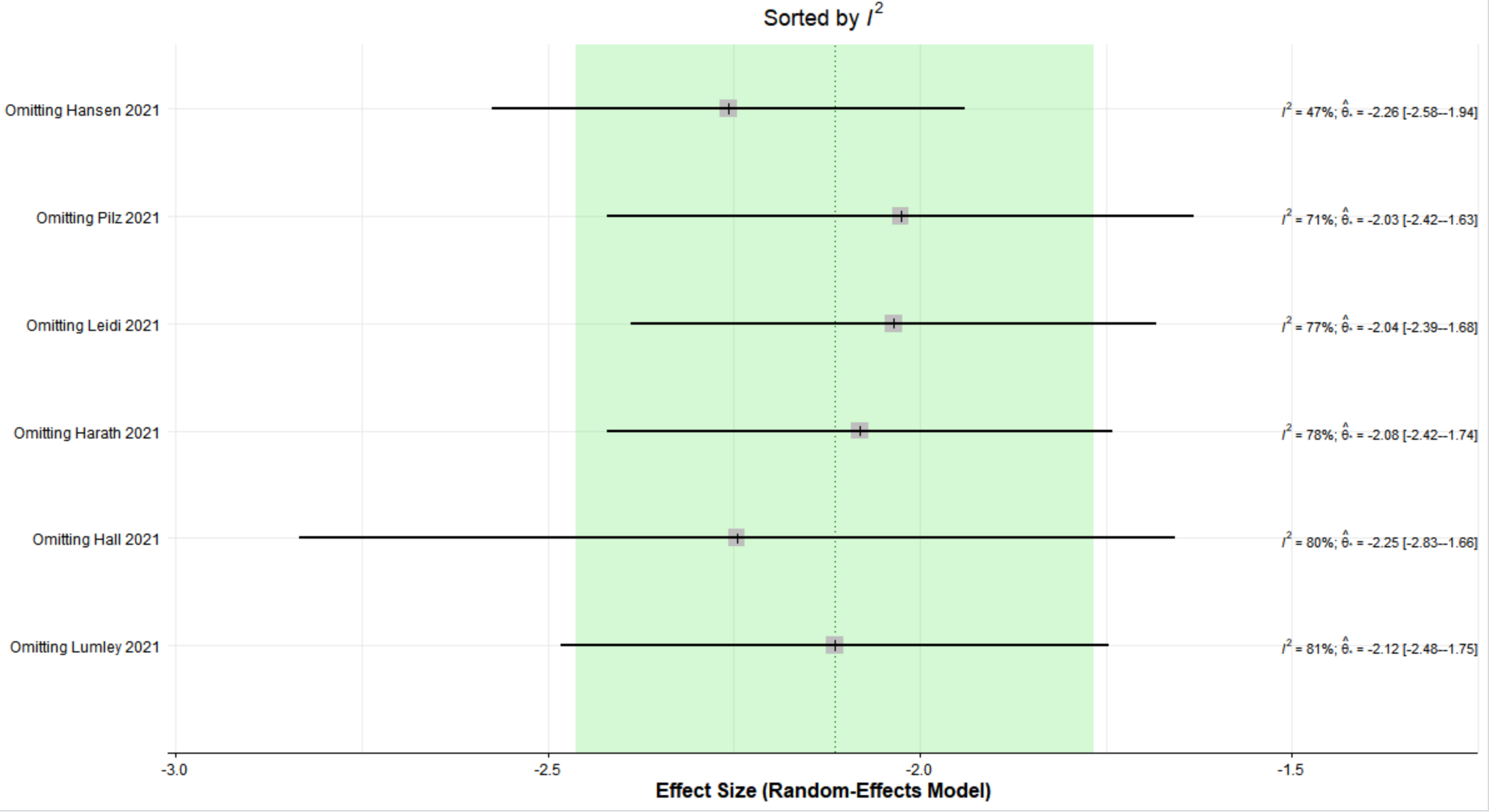

Supplement: Supplementary file 2 — Supplementary Information 2. [file 41598_2022_24220_MOESM2_ESM.png]
